# Viscous hydrophilic injection matrices for serial crystallography

**DOI:** 10.1107/S2052252517005140

**Published:** 2017-05-05

**Authors:** Gabriela Kovácsová, Marie Luise Grünbein, Marco Kloos, Thomas R. M. Barends, Ramona Schlesinger, Joachim Heberle, Wolfgang Kabsch, Robert L. Shoeman, R. Bruce Doak, Ilme Schlichting

**Affiliations:** a Department of Biomolecular Mechanisms, Max Planck Institute for Medical Research, Jahnstrasse 29, Heidelberg 69120, Germany; b Genetic Biophysics, Department of Physics, Freie Universität Berlin, Arnimallee 14, Berlin 14195, Germany; c Experimental Molecular Biophysics, Department of Physics, Freie Universität Berlin, Arnimallee 14, Berlin 14195, Germany

**Keywords:** high-throughput serial crystallography, room-temperature crystallography, microcrystal injection, XFEL, high-viscosity extrusion

## Abstract

Serial (femtosecond) crystallography at synchrotron and X-ray free-electron laser (XFEL) sources distributes the absorbed radiation dose over all crystals used for data collection and therefore allows measurement of radiation damage prone systems, including the use of microcrystals for room-temperature measurements. Serial crystallography relies on fast and efficient exchange of crystals upon X-ray exposure, which can be achieved using a variety of methods, including various injection techniques. The latter vary significantly in their flow rates – gas dynamic virtual nozzle based injectors provide very thin fast-flowing jets, whereas high-viscosity extrusion injectors produce much thicker streams with flow rates two to three orders of magnitude lower. High-viscosity extrusion results in much lower sample consumption, as its sample delivery speed is commensurate both with typical XFEL repetition rates and with data acquisition rates at synchrotron sources. An obvious viscous injection medium is lipidic cubic phase (LCP) as it is used for *in meso* membrane protein crystallization. However, LCP has limited compatibility with many crystallization conditions. While a few other viscous media have been described in the literature, there is an ongoing need to identify additional injection media for crystal embedding. Critical attributes are reliable injection properties and a broad chemical compatibility to accommodate samples as heterogeneous and sensitive as protein crystals. Here, the use of two novel hydro­gels as viscous injection matrices is described, namely sodium carb­oxy­methyl cellulose and the thermo-reversible block polymer Pluronic F-127. Both are compatible with various crystallization conditions and yield acceptable X-ray background. The stability and velocity of the extruded stream were also analysed and the dependence of the stream velocity on the flow rate was measured. In contrast with previously characterized injection media, both new matrices afford very stable adjustable streams suitable for time-resolved measurements.

## Abstract 2

High-viscosity extrusion injection is an efficient means for high-throughput crystal delivery for serial measurements at synchrotrons and XFELs. Hydro­gels for crystal embedding are described that are compatible with a wide range of crystallization precipitants, result in low X-ray background and afford very stable streams suitable for time-resolved measurements.

## 1. Introduction   

X-ray free-electron lasers (XFELs) provide coherent X-ray pulses of femtosecond duration and a peak brilliance that exceeds that of third-generation synchrotron sources by nine orders of magnitude. These unique X-ray beam properties significantly extend the possibilities of macromolecular crystallography by ‘outrunning’ most radiation damage effects (Chapman *et al.*, 2011 ▸; Boutet *et al.*, 2012 ▸). They thereby also reduce the required crystal size for diffraction data collection, which in turn facilitates reaction initiation for time-resolved experiments. Moreover, XFELs expand the possibilities for time-resolved experiments by increasing the attainable timescale from picoseconds to femtoseconds (Barends *et al.*, 2015 ▸; Pande *et al.*, 2016 ▸). The high intensity and short duration of XFEL pulses not only enables novel science but also requires new data-collection approaches. Exposure to the un­attenuated XFEL beam destroys all or part of the illuminated crystal as it is diffracting (‘diffraction before destruction’; Neutze *et al.*, 2000 ▸). Therefore a new crystal, or a fresh part thereof, is needed for each subsequent exposure. Accordingly, a serial data-collection approach is necessary, with each femtosecond exposure yielding a ‘still image’, a narrow slice through reciprocal space. Complete coverage of reciprocal space is typically obtained by collecting a large number of diffraction patterns of randomly oriented (micro)crystals, followed by merging of many partial intensities (Kirian *et al.*, 2010 ▸; White *et al.*, 2012 ▸; Hattne *et al.*, 2015 ▸; Kabsch, 2014 ▸).

Serial femtosecond crystallography (SFX) approaches for data collection thus rely on efficient sample exchange. In the case of microcrystals, this can be achieved by dispensing crystals on chips (Roedig *et al.*, 2015 ▸, 2016 ▸) or tapes (Roessler *et al.*, 2016 ▸) that are then rastered through the X-ray beam. Alternatively, crystals in microjets can be injected into the FEL X-ray beam (Weierstall *et al.*, 2014 ▸, 2012 ▸; Botha *et al.*, 2015 ▸; Sierra *et al.*, 2012 ▸). The latter approach comes in different flavours. To date, most SFX experiments at XFELs have relied on microcrystal delivery based on gas dynamic virtual nozzle (GVDN) injection (Weierstall *et al.*, 2012 ▸), which delivers the crystals within their mother liquor in a thin (3–5 µm diameter) liquid jet. A surrounding sheath of helium gas not only focuses the liquid column but also prevents it freezing when injecting into a vacuum. A major disadvantage of GVDN-based injectors, however, is their large sample consumption. The liquid microjet flows continuously at high flow rates and speeds (typical flowrates are 10–30 µl min^−1^, jet speed > 10 m s^−1^), while the pulsed FEL allows only sparse sampling [typical repetition rates of current XFELs are 10–120 Hz (Emma *et al.*, 2010 ▸; Ishikawa *et al.*, 2012 ▸)]. Hence most of the injected sample is never probed by the XFEL beam. This inefficiency can be reduced significantly by injecting crystals embedded in a highly viscous material (Weierstall *et al.*, 2014 ▸; Botha *et al.*, 2015 ▸). Lipidic cubic phase (LCP) injectors (Weierstall *et al.*, 2014 ▸) or high-viscosity extrusion (HVE) (Botha *et al.*, 2015 ▸) injectors have very low flow rates (0.02–2.5 µl min^−1^, stream velocity 0.05–4 mm s^−1^), allowing most if not all of the sample to be probed by the XFEL pulses. With these injectors the sample displacement can be slow enough, in fact, to allow for serial data collection at synchrotron sources (Botha *et al.*, 2015 ▸; Nogly *et al.*, 2015 ▸), where typical exposure times using PILATUS detectors are 40–100 ms.

High-throughput serial data-collection approaches, along with the required sample-delivery and data-analysis methods, developed as a predicated necessity at XFEL sources. However, since serial data collection conveniently mitigates the effects of radiation damage by distributing the dose over all crystals used for data collection, this technology is now being ported to synchrotron sources. One vital application is room-temperature data collection, in particular when using small crystals. Serial data collection using high-viscosity microstream injection approaches (Weierstall *et al.*, 2014 ▸; Botha *et al.*, 2015 ▸) is highly attractive due to its low sample consumption and the possibility of performing the experiments either in a vacuum or at atmospheric pressure. The approach is relatively straightforward for membrane protein crystals grown in the mesophase LCP (Caffrey & Cherezov, 2009 ▸), since the growth medium itself is already a suitable injection medium. However, the use of LCP for crystals not obtained by *in meso* crystallization is limited, due to compatibility and stability issues of commonly used crystallization solutions [*e.g.* a high concentration of ammonium sulfate or 2-methyl-2,4-pentanediol (MPD)] with the currently available lipids used for LCP formation. Moreover, for membrane protein crystals grown *in surfo*, the concentration of detergent surrounding the protein molecules can decrease as the detergent partitions into the LCP, which may destabilize the protein and crystals (Fromme *et al.*, 2015 ▸). Efforts have therefore been made to identify alternative high-viscosity media for crystal embedding. Hydro­phobic media, such as grease (Sugahara *et al.*, 2015 ▸) and Vaseline (Botha *et al.*, 2015 ▸) are very versatile: they are compatible with most crystallization conditions since they form emulsions, with the crystals being contained in tiny droplets of mother liquor surrounded by carrier media. The latter, however, results in relatively high background scattering and is often poorly suited for membrane proteins.

The high background scattering and incompatibility with membrane proteins of hydro­phobic media have motivated efforts to identify hydro­philic viscous carrier media that might not suffer from these drawbacks as they are mostly composed of water. However, in general these are difficult to identify, because they often lack the required high viscosity, especially when mixed with commonly used crystallization solutions. So far, agarose (Conrad *et al.*, 2015 ▸) and hyaluronic acid (Sugahara *et al.*, 2016 ▸) have been described, but neither is universally applicable. Thus, there is a continuing need to identify additional hydro­philic viscous media with reliable injection properties and a broad chemical compatibility to accommodate samples as heterogeneous and sensitive as protein crystals. Here, we present the use of two hydro­philic gelling polymers, sodium carb­oxy­methyl cellulose and a thermo-reversible block polymer, Pluronic F-127, as viscous injection matrices for serial crystallography. We characterize their general chemical compatibility with commonly used crystallization precipitants, as well as their injection properties, in particular stable and reproducible streaming coupled with an adjustable and stable injection speed. This is particularly important for time-resolved measurements. To determine the suitability of the two media for serial diffraction experiments, we used three model systems to cover typical crystallization conditions: glucose isomerase (which crystallizes from ammonium sulfate), thermolysin [crystallized from poly­ethyl­ene glycol (PEG)] and lysozyme (crystallized from high-salt low-pH conditions). In addition, using bacterio­rhodopsin crystals, we show that Pluronic F-127 is a useful and convenient additive to stabilize the injection properties of LCP. It also promotes LCP formation if transition to an undesired birefringent mesophase occurs during crystallization. This prevents the strong diffraction from the mesophase’s powder rings, similar to the addition of 7.9 MAG (monoacylglycerol) in the case of data collection *in vacuo* (Weierstall *et al.*, 2014 ▸).

## 2. Materials and methods   

### 2.1. Crystal preparation   

Lyophilized thermolysin (TRL) from *Geobacillus stearo­thermophilus* (No. P1512, Sigma) was dissolved in 50 m*M* NaOH at a concentration of 25 mg ml^−1^ and crystallized as described earlier (Hattne *et al.*, 2015 ▸), except that seeding was used to control crystal size. Crystals were grown by batch approaches in 1.5 ml microtubes. 200 µl protein and 200 µl precipitant (40% PEG 2000, 0.1 *M* MES pH 6.5, 5 m*M* CaCl_2_ containing crystal seeds) solutions were mixed rapidly. Crystals reached their final size (50–130 × 5–10 × 5–10 µm) overnight. After settling to the bottom of the microtube, they were washed with storage solution (20% PEG 2000, 0.1 *M* MES/NaOH pH 6.5, 5 m*M* CaCl_2_).

Crystalline glucose isomerase (GI) suspension was purchased from Hampton Research (HR7-102), repeatedly dialysed against 10 m*M* HEPES/NaOH pH 7.0, 1 m*M* MgCl_2_, and filtered through a 0.22 µm filter. Crystals were prepared by rapidly mixing 200 µl of protein (80 mg ml^−1^) and 200 µl of precipitant [2.6 *M* (NH_4_)_2_SO_4_, 0.1 *M* Tris–HCl pH 7.5, 1 m*M* MgCl_2_, containing crystal seeds] solutions. Spherical crystals (10–15 × 10–15 × 10–15 µm) grew overnight. After settling to the bottom of the microtube, the crystals were washed several times with storage solution [1.4 *M* (NH_4_)_2_SO_4_, 0.1 *M* Tris–HCl pH 7.5, 1 m*M* MgCl_2_].

Hen egg-white lysozyme (HEWL) crystals (30 × 20 × 20 µm) were prepared as described by Botha *et al.* (2015 ▸). Briefly, 2.5 ml protein solution (30–60 mg ml^−1^) was mixed with 7.5 ml precipitant solution [20% (*w*/*v*) NaCl, 6% (*w*/*v*) PEG 6000, 0.1–0.5 *M* HOAc/NaOH pH 3.5] and incubated overnight at room temperature. After settling of the crystals, the crystallization solution was exchanged several times for storage solution [8% (*w*/*v*) NaCl, 0.1 *M* HOAc/NaOH pH 4.0].

Bacteriorhodopsin-rich purple membranes from *Halobacterium salinarum* were expressed and isolated as described by Gordeliy *et al.* (2003 ▸). Purification and crystallization (Nogly *et al.*, 2015 ▸) were performed in the dark or under dim red light. The protein concentration was determined spectrophotometrically at 560 nm, using an extinction coefficient of 63 000 *M* ^−1^ cm^−1^. Purple membranes [0.9 mg ml^−1^ bacterio­rhodopsin (bR)] were mixed with 1.7% (*w*/*v*) *n*-octyl-β-d- glucoside (β-OG; Anagrade, Anatrace), 50 m*M* K_2_HPO_4_/NaH_2_PO_4_ pH 6.9, and sonicated and solubilized for 24 h. The pH was adjusted to 5.5 prior to ultracentrifugation (Ti70, 50 000 rpm, 4°C, 30 min) and the supernatant was subjected to size-exclusion chromatography (HiLoad 16/600 Superdex 75 pg, GE Healthcare) in 25 m*M* K_2_HPO_4_/NaH_2_PO_4_ buffer pH 5.6, 1.2% β-OG. The bR eluting in the second peak was concentrated to 35–50 mg ml^−1^ for crystallization in LCP using monoolein (NuChek Prep) and gas-tight Hamilton syringes (1710 or 1725 RN) connected with a coupler (TTP Labtech). A string of LCP was extruded into a Hamilton syringe filled with precipitant (30% PEG 2000, 0.1 *M* K_2_HPO_4_/NaH_2_PO_4_ pH 5.6), as described previously (Nogly *et al.*, 2015 ▸; Liu *et al.*, 2014 ▸). Very densely packed purple hexagonal plate-shaped bR crystals (20–50 µm wide and a few micrometres thick) appeared in a strongly birefringent mesophase within days.

### 2.2. Gel preparations and embedding   

A gel is a bicoherent system in which a dispersed solid phase is completely interpenetrated by a continuous liquid phase (Junginger, 1994 ▸). Specifically, a hydro­gel is a cross-linked three-dimensional network of a hydrated polymer dispersed in water. Gel preparation often exploits the temperature-dependant solubility of polymers. Some polymers have a higher solubility in hot water and are therefore heated during preparation, either to boiling (agar, agarose) or below (gelatine, sodium carb­oxy­methyl cellulose). Other polymers are more soluble in cold water and so are prepared therein (methyl­cellulose, poloxamers). Particularly for preparations of highly viscous gels, a frequent undesired feature is that clumps of powdered/flaked polymer easily form once in contact with water. The surface of the clumps rapidly hydrates (swells), which impedes water penetration into the core. Nevertheless, eventually the clumps do slowly and spontaneously disappear. Due to the increased viscosity, vigorous stirring/vortexing at this stage may not be possible but in any event is not helpful, as it incorporates undesired bubbles into the gel, which again take time to dissipate. Consequently, preparing a highly viscous gel can take up to two or three days and is largely an impromptu process. Importantly, the swelling of a polymer and the resulting viscosity of the gel depend to a varying extent on the solutes in the aqueous solution. In our experience better results are achieved when a stock gel is prepared in water and post-swelling mixed with a solution of 1–2× the desired final concentration instead of preparing the gel directly in a 1× solution.

Care was taken to prepare the gels to be as dust-free as possible, to minimize the risk of clogging problems during injection and to prevent microbial contamination, as some gels can serve as growth media. All solutions used for preparations were filtered with a 0.22 µm filter, and sterile plastic ware was used when possible.

#### 2.2.1. Carb­oxy­methyl cellulose sodium salt   

Carb­oxy­methyl cellulose sodium salt ultra-high viscosity (NaCMC; No. 21904, Sigma) forms a rigid gel in water starting at 2.5% (*w*/*v*) concentration and the viscosity increases with concentration until the solubility limit is reached, which lies slightly above 10% (*w*/*v*) concentration. For our purposes, a 7% (*w*/*v*) stock gel was prepared by sprinkling 7 g of NaCMC over 100 ml ultra-pure water in a 600 ml beaker in order to have a large water surface coming in contact with the polymer. To prevent evaporation and dust accumulation, the beaker was sealed with Parafilm M, which was perforated with a needle 3–5 times. The mixture was heated (60–70°C) and occasionally carefully homogenized with a spatula to help uniform swelling, while minimizing the trapping of bubbles. A homogenous thick gel formed within 2 d at room temperature and was stored for weeks at 4°C to prevent the growth of microorganisms. An alternative and faster protocol is to add NaCMC slowly to vigorously stirred water in a large beaker. Once all the powder has been added, the stirring speed is decreased and the mixture heated (60–70°C) under vacuum for 1 h. Stirring and heating are subsequently switched off and the gel is left overnight in a vacuum to hydrate completely and to remove residual bubbles that may disturb injection.

Prior to embedding crystals in the gel, it was equilibrated with the crystal storage solution. To this end, the stock gel was transferred with a spatula into a 10 ml plastic syringe. Next, 5 g of the stock gel was extruded through a 4 mm diameter needle into a 25 ml beaker and 5 ml of 1× or 2× storage solution was added (1× storage solution for TRL and GI crystals, 2× storage solution for HEWL crystals). The mixture was heated for 1–2 h (60–70°C) and allowed to swell overnight. The gel equilibrated with precipitant solution was then transferred into a 10 ml plastic syringe to allow convenient filling of Hamilton syringes, which were then used to mix 45 µl of the gel evenly with 1–2.5 µl of crystal pellet using a coupler. Care was taken to avoid trapping bubbles at any stage.

#### 2.2.2. Pluronic F-127   

**Figure 1:**
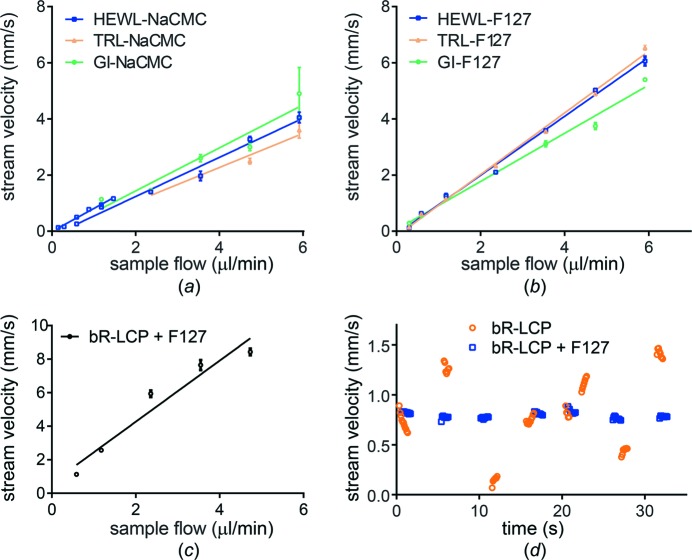
Measurements of stream velocity and its dependence on the flow rate [parts (*a*), (*b*) and (*c*)] and time [part (*d*)]. (*a*), (*b*), (*c*) The average velocities at various sample flow rates were plotted and fitted with a simple linear regression. The standard deviation is plotted for all velocity values, but it is not displayed if it is smaller than the size of the symbol. Stream velocities of (*a*) NaCMC and (*b*) F-127 as measured for different flow rates for various embedded crystals (GI, HEWL and TRL, represented by green dots, blue squares and yellow triangles, respectively). Embedded HEWL crystals in NaCMC were measured in both small and large sample reservoir injectors, hence the two blue plots covering lower and higher flow rates in part (*a*). (*c*) Stream velocity of bR-LCP mixed with F-127 (in a 3+1 ratio) measured at different flow rates. (*d*) At a constant flow rate (0.3 and 0.35 µl min^−1^ for the bR-LCP sample without and with F-127, respectively) at intervals of approximately 5 s, 5–10 instantaneous stream velocities were calculated and plotted for both samples. Data points for bR-LCP and bR-LCP with F-127 are represented as orange circles and blue squares, respectively.

Pluronic F-127 (P2443, Sigma) forms a rigid thermo-reversible gel in water at 20–35% (*w*/*w*) concentration, which is liquid at 4°C and solid at room temperature. A 35% (*w*/*w*) stock solution was prepared by adding 16 g of F-127 to 29.7 ml cold water in a 50 ml plastic tube. Only gentle mixing with a spatula is possible, as F-127 readily creates a foam. Therefore, the mixture was allowed to dissolve in a cold room and gently stirred only 1–2 times per day to mix the pelleted/clumped polymer better into the gelling liquid. This process typically took 3 d, creating a clear viscous liquid (similar to liquid honey in viscosity), which was stored in a closed tube to avoid drying out. To embed TRL, GI or HEWL crystals, the cold 35% F-127 stock solution was poured into a 5 ml plastic syringe for the transfer of 50 µl into a Hamilton syringe. This was then coupled to another Hamilton syringe filled with crystal storage solution (12.5 µl 1×, 2.5 µl 1.5× or 12.5 µl 2× in the case of TRL, GI and HEWL, respectively) and homogenized. The entire content was then moved to the syringe that originally contained the storage solution, the syringes were decoupled and the empty syringe was filled again with the same volume of storage solution. It was then reattached to the other syringe and the mixture was again homogenized. In this manner, the storage solution was added from ‘both ends’ to facilitate complete homogenization, which was confirmed by extruding a small amount of the gel through the coupler from both ends and from the middle of the coupled syringes, making sure that no droplets indicating liquid were present. Notably, once the gel was mixed with the storage solutions or with other tested chemicals (except for the TRL storage solution that contains 20% PEG 2000), the thermo-reversible properties dis­appeared and the gel was irreversibly solid, likely due to the increased ionic strength. 45 µl of the gel was then mixed with 2.5–5 µl of crystal pellets using coupled Hamilton syringes and loaded into the HVE injector. In the case of the *in meso* grown bR crystals, the crystal-containing and strongly birefringent mesophases from multiple syringes were harvested and pooled. For the diffraction experiment, 25 µl of this mesophase were mixed with 25 µl of 35% F-127, yielding a transparent phase within minutes. For measurements of stream velocity as a function of flow rate (Fig. 1 ▸ *c*) or time (Fig. 1 ▸ *d*), F-127 was mixed with LCP at ratios of 1+3 and 1+2 by volume, respectively. A similar Pluronic compound, Pluronic F-108 (No. 542342, Aldrich), prepared as a 40% stock gel, can be mixed with LCP in an analogous manner.

#### 2.2.3. Previously described media   

The synthetic grease Super Lube (No. 21030, Synco Chemical Co.) was mixed with crystals as described by Sugahara *et al.* (2015 ▸). Briefly, 95 µl of grease and 5 µl of crystal pellets were mixed with a spatula on a glass slide and transferred to a 250 µl Hamilton syringe using the spatula. A hydro­philic matrix consisting of 5.6% (*w*/*v*) ultra-low gelling agarose (No. A5030, Sigma) and 30% glycerol (Conrad *et al.*, 2015 ▸) was prepared by heating to 100°C using a ThermoMixer C (Eppendorf). Hyaluronic acid (No. H5388, Sigma) was prepared as a 12% (*w*/*v*) aqueous mixture (Sugahara *et al.*, 2016 ▸).

### 2.3. Compatibility tests   

The precipitant solutions for the compatibility tests were: 2.5 *M* Li_2_SO_4_, 2.5 *M* MgSO_4_, 3.6 *M* (NH_4_)_2_SO_4_, 4 *M* NaCl, 70% (*w*/*v*) PEG, 50% (*w*/*v*) PEG 2000, 50% (*w*/*v*) PEG 4000, 50% (*w*/*v*) polypropyl­ene glycol (PPG) 400, 80% (*v*/*v*) ethanol, 50% (*v*/*v*) 2-methyl-2,4-pentane­diol (MPD). If needed, the pH of PEGs, PPG and salts was adjusted to neutral with 1 *M* Tris–HCl pH 7.5.

Carb­oxy­methyl cellulose gel equilibrated with the precipitant solution was prepared by mixing 2 g of 7% (*w*/*v*) stock NaCMC gel (as described in Section 2.2.1) with 2 ml of precipitant solutions listed above. To estimate homogeneity, viscosity and injection properties visually, the mixture was manually extruded from a Hamilton syringe through a 410 µm inner diameter (ID) needle.

To test the compatibility of 35% F-127, 100 µl were mixed with 50 or 100 µl of the precipitant solutions listed above using two coupled glass syringes. Homogeneity, viscosity and injection properties were visually estimated by manually extruding the mixture through the coupler (410 µm ID).

### 2.4. Stream velocity measurements   

To determine the velocity of the extruding stream, movies of the stream were recorded at 50–60 Hz with a high-speed camera (Photron FASTCAM SA-Z equipped with a Navitar 12× zoom lens, 2× adapter and 2× lens attachment) for a chosen stream flow rate (0.3–5.9 µl min^−1^). In the movies, a feature in the extruded stream (*e.g.* a crystal) was tracked with time as it moved downstream. Length scales in the movie image were explicitly calibrated against a standard calibration slide and the frame speed of the camera was very well defined. Accordingly, the stream velocity could be computed very accurately by measuring the displacement of the tracked feature from the nozzle tip from frame to frame. For measurements of stream velocity as a function of flow rate (Figs. 1 ▸ *a*, 1 ▸ *b* and 1 ▸ *c*), 3–6 of these points were averaged to obtain an average velocity value at a given flow rate. For measurements of stream velocity as a function of time (Fig. 1 ▸ *d*), the flow rate was kept constant at 0.3 or 0.35 µl min^−1^ for the bR–LCP samples without and with F-127, respectively. At intervals of approximately 5 s a feature was identified and tracked as it moved with the extruded stream. Its increasing displacement from the nozzle tip was measured over time in 80–120 ms increments and stream velocities were calculated for 5–10 of these individual measurements.

### 2.5. Serial crystallography data collection at a synchrotron   

#### 2.5.1. Sample injection and data collection at PXII   

The sample reservoir of our HVE injector (Botha *et al.*, 2015 ▸) was loaded directly from Hamilton 1710 RN syringes by means of a custom loading jig. The HVE injector was mounted on the goniometer head of the X10SA (PXII) beamline at the Swiss Light Source (SLS), pointing vertically downward to benefit from gravitational force on the extruding stream (Botha *et al.*, 2015 ▸). The IDs of the capillaries were chosen according to crystal size and desired stream velocity: for F-127 and NaCMC samples, 100 and 150 µm ID capillaries were used, respectively. For comparison data sets in grease, the same ID capillary was used as for the sample under comparison. Sample consumptions and stream velocities are listed in Table 1 ▸. By means of a Shimadzu 20AD HPLC (high-performance liquid chromatography) pump amplified by the intrinsic piston factor of the HVE injector (Botha *et al.*, 2015 ▸), a pressure of 350–700 psi (1 psi ≃ 6893 Pa) was applied to the sample. The pressure of the guiding helium-sheath gas was typically 5–15 psi. Still diffraction data (no oscillation) were collected at room temperature in the high-flux undulator setting using the full beam (3.4 × 10^12^ photons s^−1^ in 50 × 10 µm) and a wavelength of 1.033 Å, except for the data for HEWL in grease, which were collected at 0.954 Å (2.4 × 10^12^ photons s^−1^ in 50 × 10 µm). The PILATUS 6M detector was operated in a continuous shutterless mode and still diffraction patterns were recorded at 10 Hz (exposure time 100 ms).

#### 2.5.2. Data processing   

The diffraction data were processed with *nXDS* (Kabsch, 2014 ▸). The program uses an Ewald offset correction factor to estimate reflection intensities recorded on still exposures and implements post-refinement to improve all diffraction parameters and scaling and correction factors (Kabsch, 2014 ▸). *nXSCALE* was used for scaling of data streams from the same sample but collected with different parameters. Background profiles were calculated by determining the median intensity in all pixels over 50 evenly spaced images of a data set. The resulting background images were then radially integrated after applying a beamstop mask.

## 3. Results and discussion   

Serial crystallography requires a continuous supply of crystalline material to the interaction zone with the X-ray beam. This is frequently achieved using either a GDVN injector (Weierstall *et al.*, 2012 ▸) that produces a fast liquid microjet of protein crystals in their mother liquor, or an HVE injector (Weierstall *et al.*, 2014 ▸; Botha *et al.*, 2015 ▸) that slowly extrudes a stream of crystals embedded in a viscous medium. The latter offers significantly reduced sample consumption due to the low speed of the stream, which moreover can be matched to the XFEL repetition or detector frame rate. This sample-delivery technique is therefore suitable for data collection not only at XFELs but also at synchrotron sources, a major advantage given the scarcity of XFEL beamtime. HVE injection, however, also has drawbacks compared with GDVN-based injection: the diameter of a viscous stream is significantly larger than that of a liquid microjet, resulting in higher X-ray background and thus requiring larger crystals; the flow stability of a viscous stream is lower, which can render time-resolved experiments more complex if not impossible; and last but not least only a few viscous injection carrier media have been described so far (Sugahara *et al.*, 2015 ▸, 2016 ▸; Conrad *et al.*, 2015 ▸; Botha *et al.*, 2015 ▸). This severely limits the current choice of suitable matrix materials for crystal embedding, which can be problematic given the high sensitivity of most protein crystals. The aforementioned drawbacks of HVE injection are related to the viscous matrix material used. Thus, to reduce these disadvantages and to exploit fully the potential of HVE injection as a low sample consumption and high-throughput sample-delivery technique, more viscous injection matrices need to be identified and, importantly, well characterized in their key properties. These include: (i) compatibility with macromolecular crystals and their crystallization solutions; (ii) visco-elastic properties supporting the formation of a stable stream upon extrusion; (iii) low X-ray background and no impact on the diffraction quality of crystals; and (iv) compatibility with the desired experimental setup, which can require high or low flow rates, be in a vacuum or at ambient pressure *etc*. When searching for suitable compounds that fulfil these requirements, we focused primarily on gelling high molecular weight polymers that are proven to be compatible with biological materials [fulfilling requirements (i) and (ii)], either because they are already used in macromolecular crystallography (Sugiyama *et al.*, 2013 ▸) or because they are used in the food industry or pharmaceutical formulations (Saha & Bhattacharya, 2010 ▸).

Thermo-reversible hydro­gels such as Mebiol (Sugiyama *et al.*, 2013 ▸) are liquid at low temperature and solid at room temperature; they are therefore attractive embedding mater­ials as we described earlier (Botha *et al.*, 2015 ▸). Here we focus on poloxamers, a related copolymer series of thermoreversible hydro­gels composed of triblocks of PEG_(*x*)_–PPG_(*y*)_–PEG_(*z*)_ with the trade name Pluronic (Schmolka, 1972 ▸). In particular, the F-127 compound has been used for *e.g.* crystallization (Cespi *et al.*, 2012 ▸), treatment of burns (Schmolka, 1972 ▸) and pharmaceutical formulations (Escobar-Chávez *et al.*, 2006 ▸). This last field, together with the food industry, also heavily employs a cellulose derivative, carb­oxy­methyl cellulose sodium salt (NaCMC) as a thickening and gelling agent (Hollabaugh *et al.*, 1945 ▸; Saha & Bhattacharya, 2010 ▸). We tested whether F-127 and NaCMC fulfil the aforementioned required properties (i)–(iv) of injection matrices for serial crystallography using both vapour-diffusion and *in meso* grown protein crystals. We present a detailed characterization of these two previously undescribed injection matrices.

### 3.1. Chemical compatibility   

Hydro­philic injection matrices are miscible with protein crystallization precipitants, but mixing can alter their visco-elastic properties critical for injection and change their solubility, resulting even in precipitation. Therefore, vigorous testing of matrix–precipitant compatibility is an essential first characterization of any hydro­philic injection matrix. We first determined the concentrations of NaCMC and F-127 needed for the formation of a sufficiently viscous matrix suitable for injection [2.5% (*w*/*v*) for NaCMC, 20% (*w*/*w*) for F-127 in water]. We next prepared a highly concentrated stock gel of each compound [7% (*w*/*v*) for NaCMC, 35% (*w*/*w*) for F-127]. The stock gels were then mixed with a selection of common crystallization reagents including salts, polymers and organic solvents. If the resulting mixture was chemically compatible (homogenous, viscous, translucent, without precipitated material), it was subjected to a simplified injection test comprising manual extrusion through a 410 µm ID needle. The observed injection properties were visually judged as good (+) or very good (++) depending on the stability and viscosity of the extruded material (viscous enough to form and maintain a continuous stream uninterrupted by droplets or inhomogeneity). Next, we embedded protein crystals in each of the two media equilibrated with the corresponding crystal-storage solutions and checked under a light microscope whether the embedded crystals were stable (no cracks, no dissolution) for at least 2 h. For the *in meso* grown bR crystals, the gel was mixed directly with the harvested crystal-loaded LCP that contained only residual quantities of the crystallization precipitant which were readily absorbed by the gel.

NaCMC displays a very broad chemical compatibility and forms a stable stream even at high concentrations of the tested crystallization precipitants (Table 2 ▸). Embedded GI, TRL and HEWL crystals in NaCMC equilibrated with storage solutions were inspected under a light microscope and looked intact (clear sharp edges) for at least 6 h after embedding.

F-127, in contrast with NaCMC, tolerates only NaCl and low molecular weight PEG/PPG at high concentration. Other compounds, such as (NH_4_)_2_SO_4_, are compatible only at lower concentrations (Table 3 ▸). Crystals of GI, TRL and HEWL in F-127 equilibrated with the appropriate storage solution appeared visually intact (clear sharp edges) for at least 2 h after embedding. The gelling properties of both media are not affected by the addition of the detergent β-OG at 1% (*w*/*v*), indicating that the medium could be used with membrane protein crystals obtained from vapour-diffusion crystallization. The birefringent mesophase with grown bR crystals quickly became transparent upon mixing with F-127 and crystals remained in a non-birefringent phase for at least 18 h.

When comparing the chemical compatibility of NaCMC and F-127 with those of established hydro­philic injection matrices, NaCMC and F-127 can tolerate higher concentrations of NaCl (2 *M*) than agarose (1 *M*). Moreover, NaCMC is also compatible with higher concentrations of (NH_4_)_2_SO_4_ (1.8 *M*) than agarose (1.25 *M*) (Conrad *et al.*, 2015 ▸). The compatibilities of NaCMC and F-127 with PEGs and organic solvents are listed in Tables 2 ▸ and 3 ▸ but unfortunately they cannot be directly compared with agarose as no detailed information was provided on the concentrations used in the latter case (Conrad *et al.*, 2015 ▸). Likewise, only limited comparisons can be made with hyaluronic acid (Sugahara *et al.*, 2016 ▸), as it was only equilibrated with a crystallization solution [presumably final concentration 7% (*w*/*v*) sodium chloride, 2% (*w*/*v*) PEG 6000 and 0.05 *M* sodium acetate (pH 3.0)] of lysozyme and water in the case of proteinase K crystals. No further precipitant compatibilities were described, possibly due to the very high cost of the compound.

Many crystallization conditions contain high salt concentrations that are often poorly compatible with established hydro­philic viscous embedding matrices [Mebiol, agarose, high molecular weight PEGs, hyaluronate or polyvinyl­pyrrolidones (PVPs)]. Interestingly, many hydro­philic gelling agents that tolerate high electrolyte concentrations and/or low pHs are used in the food industry (Saha & Bhattacharya, 2010 ▸). Using this information, we screened a number of these compounds and, based on preliminary results, we further investigated the use of the following compounds as injection matrices at the specified concentrations: xanthan [4% (*w*/*v*)], guar [2% (*w*/*v*)] in combination with xanthan [2% (*w*/*v*)], and tragacanth [3% (*w*/*v*)]. They form viscous gels when dissolved in *e.g.* 2.1 *M* K_2_HPO_4_/NaH_2_PO_4_ and afford stable streams upon extrusion, thus providing a good starting point for samples requiring high salt concentrations. In contrast, gellanum gum and κ-carrageenan are likely of only limited use as injection matrices since these compounds form rather brittle gels.

### 3.2. Injection   

The formation of a stable stream upon extrusion is another critical requirement for a useful injection matrix. The stream needs to run continuously, both mechanically (no bubbles, liquid droplets or other breaks) and temporally, *i.e.* with constant velocity. The latter has two aspects. First, in general, a stable and appropriate stream velocity ensures that sample exposed to and subsequently damaged by the X-ray beam proceeds out of the interaction zone before the next X-ray exposure ensues at the chosen data-collection rate (*f*). Second, time-resolved experiments, for example using an optical pump–X-ray probe scheme for data collection, require in addition that probed crystals be activated only by the pump pulse that preceded the probe pulse by a desired time delay (Δ*t*) and that these pumped crystals clear the X-ray probe region before the next probe pulse arrives (Δ*t* + 1/*f*). This requirement sets a minimum stream velocity for a given *f*. In addition, a maximum velocity limit is set by the requirement that the pumped crystal has not moved out of the X-ray probe region before the associated pump pulse arrives at Δ*t*. This is particularly critical in the case of long time delays (Δ*t* in the millisecond range) and high repetition rates (*f* in the 60–120 Hz range, with 1/*f* > Δ*t*). In the ideal case, data collection is performed at the maximum possible repetition rate *f* for maximum time efficiency and the velocity of the stream is adapted accordingly by adjusting the flow rate. However, if the extruded stream is stable only within a narrow range of velocities, the repetition rate needs to be adjusted. Thus, knowledge of the stability of the stream velocity and its dependence on flow rate is important. So far, these parameters have been largely disregarded (Nogly *et al.*, 2016 ▸). Previous characterizations of injection media described in more or less detail their chemical compatibility and X-ray background based on serial data collection at synchrotron (Botha *et al.*, 2015 ▸) or XFEL (Sugahara *et al.*, 2015 ▸, 2016 ▸; Conrad *et al.*, 2015 ▸) sources, but not their flow properties. For instance, in our experience the grease matrix (Sugahara *et al.*, 2015 ▸) has very unstable injection properties (see supplementary Fig. S1), affecting the efficient use of beam time and sample and complicating time-resolved experiments.

Therefore, to obtain this important information on the injection properties of the different media, we examined the dependence of the stream velocity (in mm s^−1^) on the flow rate (in µl min^−1^) of the NaCMC and F-127 matrices with embedded crystals to judge their suitability for time-resolved experiments by identifying the range of stable stream velocities. Importantly in regard to experiments at XFELs, injection with no or small embedded crystals (<10 µm) is typically more stable and smaller ID nozzles (*e.g.* 50 µm) can be used for both media, whereas injection with larger crystals (>20 µm) requires larger ID nozzles (100 µm for F-127 and 150 µm for NaCMC) for stable extrusion. We recorded movies of the streams extruding at given flow rates and calculated their velocities by measuring the distances that given particles travelled within a certain time frame. The measurements were performed at ambient pressure in air using our HVE injector. In our experience, injection at ambient pressure is more challenging than in a vacuum, since the latter seems to prevent attachment of extruding material to the nozzle tip (a common problem with grease injection, see supplementary Fig. S1). On the other hand, evaporative cooling of the extruded material *in vacuo* can hamper data collection. We observed dehydration of both NaCMC and F-127 when injected into a vacuum (8.6 mbar; 1 bar = 100 000 Pa), which may be prevented by the addition of stabilizers or cryo­protectants. For example, F-127 mixed with LCP (volume ratio 1+3) can be injected into a vacuum without freezing problems.

#### 3.2.1. Carb­oxy­methyl cellulose sodium salt (NaCMC)   

NaCMC forms stable streams with a wide range of precipitants. As an injection matrix, however, NaCMC is particularly sensitive to the size of embedded crystals, with larger crystals notably disturbing the stream stability. In order to extend the feasibility of NaCMC use into this regime of larger crystals such as in our experiment at the SLS, we optimized the injection conditions for this challenging case by applying the following measures: (i) decreasing the crystal concentration [from 5% (*v*/*v*) to 2.5% or less]; (ii) choosing a larger ID nozzle (150 µm) (using nozzles with larger IDs typically requires lowering the crystal concentration, as the stream diameter together with the focal size of the X-ray beam define the volume that contains ideally one crystal per X-ray exposure); and (iii) decreasing the precipitant concentration if possible. In this way we achieved stable injection for all three samples (GI, HEWL and TRL; see supplementary movie S1). The velocity of the stream depends linearly on the flow rate, and the generally small standard deviation indicates that the velocity is stable (see Fig. 1 ▸ *a*). This demonstrates that flow-rate settings can indeed be used to control stream velocity over a very large range (0.13–4.90 mm s^−1^). The dependence of the stream velocity on the flow rate varies slightly between samples due to small differences in jet diameter – the larger the stream diameter, the slower the stream at a given flow rate.

The stream diameter depends on the crystal size and on the precipitant. Given this variability, it is necessary to optimize sample preparation and injection for each sample individually, starting with the three parameters listed above. Due to the stable injection properties (constant velocity and stream diameter), jet velocities can be estimated reasonably well based on the diameter of the stream and the known flow rate, thus allowing live feedback during an experiment at a synchrotron or XFEL.

#### 3.2.2. Hydro­gel Pluronic F-127   

F-127 has specific visco-elastic and textural properties (spreadability, creaminess) that support the formation of very stable streams. Precipitant, crystal size and concentration have less impact on stream stability and velocity for F-127 than for NaCMC (see Fig. 1 ▸ *b* and supplementary movie S2). The velocities showed a linear dependence on flow rate and are stable over a large range (0.12−6.53 mm s^−1^). The dependence of velocity on flow rate is nearly identical for the TRL and HEWL samples but differs slightly for the GI sample, as ammonium sulfate alters the visco-elastic properties of F-127 in a concentration-dependent manner. Importantly, F-127 can also be added post-crystallization to a crystal-loaded birefringent mesophase in order to adjust both the injection and mesophase properties or to dilute the sample in one step. This is a simpler and more viable protocol compared with using liquid paraffin (Nango *et al.*, 2016 ▸), where first the dilution or mesophase adjustment with LCP is performed and only then is the paraffin injection additive added. This not only takes time but also requires multiple mixing steps, which can damage the crystals. Indeed, the addition of F-127 to bR crystals in LCP allowed the fine control of the stream velocity by the flow rate (see Fig. 1 ▸ *c*) and stabilized the erratic flow behaviour observed for bR crystals in pure LCP (see Fig. 1 ▸ *d* and supplementary movies S3 and S4). The optimal mixing ratio of LCP and F-127 is 3+1 to 1+1 by volume. The more F-127 that is added, the thicker the resulting stream and therefore the lower its velocity.

**Figure 2:**
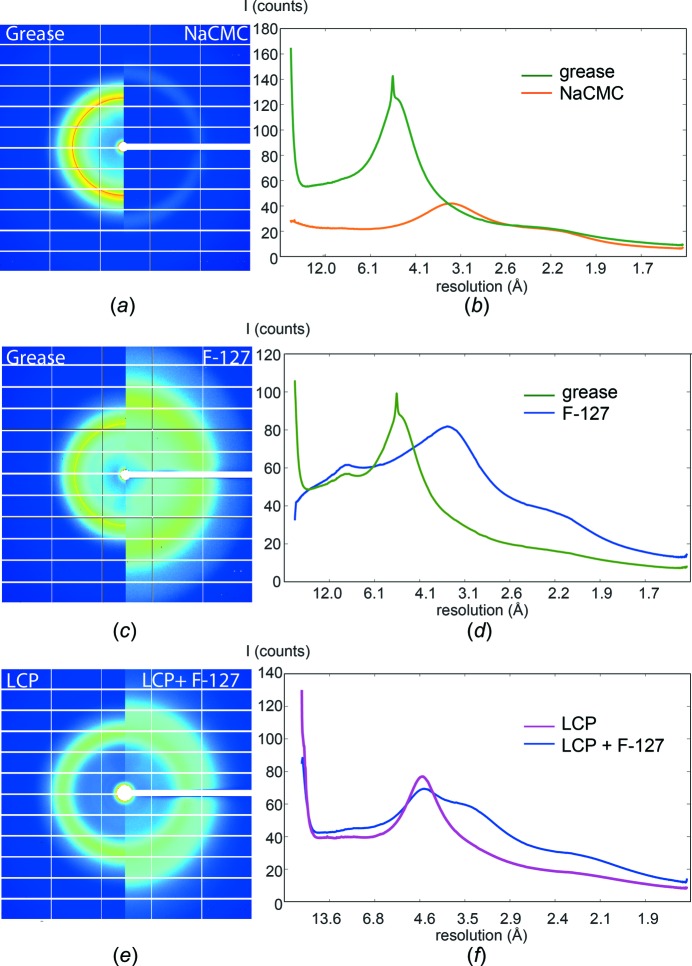
Background scattering of different media. Background scattering of (*a*), (*b*) NaCMC and (*c*), (*d*) F-127 compared with grease, and (*e*), (*f*) LCP compared with a 1:1 LCP and F-127 mixture. (*a*), (*c*), (*e*) Median background images of (*a*) NaCMC and (*b*) F-127 compared with grease and (*e*) F-127+LCP compared with LCP. Each image in parts (*a*), (*c*) and (*e*) is composed of two halves, that on the left being that of the standard medium (grease or LCP) and that on the right being that of the tested medium. (*b*), (*d*), (*f*) Radially integrated background images plotted against resolution. The green line represents grease [parts (*b*) and (*d*)], the orange line NaCMC [part (*b*)] and the blue line F-127 [part (*d*)]. In part (*f*), the purple line represents LCP and the blue line represents LCP+F-127. The following capillary diameters were used, yielding the measured stream diameters: (*a*), (*b*): capillary ID 150 µm, NaCMC diameter 220–230 µm, grease 135–145 µm; (*c*), (*d*): capillary ID 100 µm, F-127 diameter 200–230 µm, grease 80–100 µm; (*e*), (*f*): capillary ID 100 µm, LCP+F-127 diameter ∼200 µm, LCP ∼100 µm.

For comparison, we tested in our ambient-pressure injection setup using 100 µm ID nozzles the two published hydro­philic injection matrices described in the literature, hyaluronic acid (Sugahara *et al.*, 2016 ▸) and agarose (Conrad *et al.*, 2015 ▸). We could easily reproduce injection of 12% (*w*/*v*) aqueous hyaluronic acid as described by Sugahara and co-workers, who used it for XFEL data collection at ambient pressure at SACLA (SPring-8, Japan; Sugahara *et al.*, 2016 ▸). Conrad *et al.* used agarose as a carrier medium for XFEL data collection in a vacuum and at ambient pressure at the CXI endstation of LCLS at SLAC (Stanford, California, USA; Conrad *et al.*, 2015 ▸). Our experience with injection of 5.6% agarose with 30% glycerol is in line with their observation that injection at ambient pressure is much more unstable. We noticed that a stable gel stream is frequently disrupted by liquid droplets, indicating that the mixture is not homogenous, despite extensive mixing [see also Fig. 2*b* in the paper by Conrad *et al.* (2015 ▸)].

### 3.3. Serial crystallography data collection – diffraction and background   

Using our HVE injector [see Botha *et al.* (2015 ▸) for details], we performed serial crystallography experiments at the SLS on beamline X10SA (PXII) to compare NaCMC and F-127 with the standard high-viscosity embedding medium grease (Sugahara *et al.*, 2015 ▸). X-ray background and crystal diffraction quality were measured, in all cases with soluble protein crystals embedded in the carrier matrix. The combination of F-127 and LCP with membrane protein crystals of bR was compared with plain LCP with bR crystals.

NaCMC has very low background scattering with a weak diffuse ring at 2.6–4 Å. This is similar to other hydro­philic matrices [hyaluronic acid (Sugahara *et al.*, 2016 ▸) and agarose (Conrad *et al.*, 2015 ▸)] and much lower than for grease even when using a larger stream diameter (for 150 µm ID capillary: NaCMC diameter 220–230 µm; grease diameter 135–145 µm; Figs. 2 ▸ *a* and 2 ▸ *b*). The background of F-127 is comparable in magnitude with grease and LCP (for 100 µm ID capillary: F-127 diameter 200–230 µm; grease diameter 80–100 µm; Figs. 2 ▸ *c*, 2 ▸ *d*, 2 ▸ *e* and 2 ▸ *f*). The resolution range of the F-127 diffuse ring is around 2.8–5 Å and differs from grease’s diffuse ring of around 4.3–6 Å which is dominated by a strong Debye–Scherrer ring at 4.9 Å. We observed high-resolution diffraction from all our samples and collected complete data sets in 2 h using less than 0.5 mg crystalline protein for each data set. The *nXDS* analysis of a similar number of indexed diffraction patterns collected from the corresponding grease and NaCMC/F-127 embedded samples, as well as the LCP and LCP+F-127 embedded samples, yielded similar data quality (crystallographic data statistics are listed in Table 4 ▸).

The experimental setup at the SLS was well suited to our purpose of characterizing the flow properties of protein crystal-containing NaCMC and F-127 streams in the presence of X-rays, measuring the resultant X-ray background and assessing high-resolution diffraction for model systems. For the comprehensive characterization of real samples, however, addressing the following issues will be beneficial: first, using a beamline with a higher flux density will allow measurements using smaller crystals, facilitating optimization of the flow properties of the stream. The use of smaller crystals will avoid one big crystal being exposed to multiple consecutive shots as it moves through the X-ray beam, thereby possibly accumulating radiation damage (*e.g.* https://www.youtube.com/watch?v=WBhJPZixy8I; Nogly *et al.*, 2015 ▸). Moreover, using smaller crystals requires less material. Second, a higher flux density will also allow the use of detectors having a faster frame rate. This will decrease the effects of crystal rotation during exposure, particularly at higher flow rates and stream velocities. This is manifested by azimuthal rotations of the reflections in the diffraction pattern around the X-ray beam axis (Botha *et al.*, 2015 ▸). Given the availability of detectors with a shorter read-out time than the PILATUS used here [*e.g.* Eiger (Casanas *et al.*, 2016 ▸) or Jungfrau (Mozzanica *et al.*, 2014 ▸)] and numerous microfocus beamlines with higher flux density (Smith *et al.*, 2012 ▸), these issues are currently being addressed at synchrotrons. They are not complicating issues in data collection at XFELs, where the exposure time is only a few tens of femtoseconds (faster than any mechanical rotation) and the enormous XFEL photon flux already permits the use of micro- to nanometre-sized crystals.

## 4. Conclusion and outlook   

We have described two new hydro­philic injection matrices for serial crystallography. They are compatible with a wide range of crystallization precipitants and protein crystals. When injected, the loaded sample matrices deliver stable streams, have acceptable X-ray background and allow collection of high-resolution diffraction data. In particular, NaCMC has very broad precipitant compatibility, indicating a potential to accommodate many diverse crystallization conditions. F-127 has very robust injection properties with both vapour-diffusion and *in meso* grown crystals, affording very stable and tuneable stream velocities that are critical for time-resolved experiments.

High-throughput serial crystallography at XFEL or synchrotron sources, with its snapshot data-collection approach that distributes the dose over all crystals used for data collection, is an emerging technique for convenient room-temperature data collection and time-resolved experiments. Its use will become more widespread with the increased availability of suitable X-ray sources. These include several new XFEL sources that are currently being built, upgrades of third-generation synchrotrons and the commissioning of fourth-generation synchrotron sources (Eriksson *et al.*, 2014 ▸). We expect a further increase in demand for high-viscosity extrusion injection due to its low sample consumption and stream velocities. To make full use of this potential, it is important to have a broad selection of suitable and well characterized injection matrices available to accommodate many different protein crystals and experimental goals.

## Supplementary Material

Figure S1 and captions for Movies S1-S4. DOI: 10.1107/S2052252517005140/ec5001sup1.pdf

. DOI: 10.1107/S2052252517005140/ec5001sup2.mp4
Movie S1. Close-up view of the extruding stream from the HVE injector nozzle with the online microscope camera at beamline X10SA (PXII) at the SLS. The size of the X-ray beam is 50 × 10 µm. The ID of the nozzle is 150 µm and the extruding stream consists of HEWL crystals embedded in NaCMC.

. DOI: 10.1107/S2052252517005140/ec5001sup3.mp4
Movie S2. Close-up view of the extruding stream from the HVE injector nozzle with the online microscope camera at beamline X10SA (PXII) at the SLS. The size of the X-ray beam is 50 × 10 µm. The ID of the nozzle is 100 µm and the extruding stream consists of TRL crystals embedded in F-127.

. DOI: 10.1107/S2052252517005140/ec5001sup4.mp4
Movie S3. Close-up view of the extruding stream from the HVE injector nozzle with the online microscope camera at beamline X10SA (PXII) at the SLS. The size of the X-ray beam is 50 × 10 µm. The ID of the nozzle is 100 µm and the extruding stream consists of bR crystals in LCP mixed with F-127.

. DOI: 10.1107/S2052252517005140/ec5001sup5.mp4
Movie S4. Close-up view of the extruding stream from the HVE injector nozzle with the online microscope camera at beamline X10SA (PXII) at the SLS. The size of the X-ray beam is 50 × 10 µm. The ID of the nozzle is 100 µm and the extruding stream consists of bR crystals in LCP without F-127. While the diameter of the stream is fairly constant, the stream velocity is not. When the stream flows slowly, crystals (purple plates) extruding downstream can be clearly identified. When the stream accelerates, extruded crystals cannot be seen clearly and appear vertically smeared.

## Figures and Tables

**Table 1:** Selected injection parameters for different samples

Sample	Crystal size† (µm)	Nozzle ID (µm)	Flow rate (µl min^−1^)	Measured stream velocity (µm s^−1^)
bR-LCP+F-127	20–50	100	0.09	50
bR-LCP	20–50	100	0.15	Very variable
GI in F-127	10–15	100	0.15	50–60
GI in grease	10–15	100	0.06	130–290
TRL in F-127	60–130	100	0.15	60–70
TRL in grease	60–130	100	0.06–0.15	125–280
TRL in NaCMC	60–130	150	0.6	240–270
TRL in grease	60–130	150	0.15	150–170
HEWL in NaCMC	20–30	150	0.3	50–70
HEWL in grease	20–30	100	0.06–0.15	120–200

† Longest crystal dimension.

**Table 2:** Compatibility of carboxymethyl cellulose sodium salt (NaCMC) with various precipitants Injection properties were optically judged as good (+) or very good (++) depending on the stability and viscosity of the extruded stream (viscous enough to form and maintain a continuous stream not interrupted by droplets or inhomogeneities).

Compound	Type of precipitant	Final tested concentration	Injection
Lithium sulfate	Salt	1.25 *M*	+
Magnesium sulfate	Salt	1.25 *M*	+
Ammonium sulfate	Salt	1.8 *M*	+
Sodium chloride	Salt	2 *M*	+
Polyethylene glycol 400	Polymer	35% (*w*/*v*)	++
Polyethylene glycol 2000	Polymer	30% (*w*/*v*)	++
Polyethylene glycol 4000	Polymer	25% (*w*/*v*)	++
Polypropylene glycol 400	Polymer	25% (*w*/*v*)	++
Ethanol	Volatile organic liquid	35% (*v*/*v*)	+
2-Methyl-2,4-pentanediol (MPD)	Non-volatile organic liquid	25% (*v*/*v*)	+

**Table 3:** Compatibility of Pluronic F-127 with various precipitants Injection properties were optically judged as good (+) or very good (++) depending on the stability and viscosity of the extruded stream (viscous enough to form and maintain a continuous stream not interrupted by droplets or inhomogeneities).

Compound	Type of precipitant	Final tested concentration	Injection
Ammonium sulfate	Salt	0.25 *M*	+
Sodium chloride	Salt	2 *M*	++
Polypropylene glycol 400	Polymer	25% (*w*/*v*)	+
Polyethylene glycol 400	Polymer	23% (*w*/*v*)	++
Polyethylene glycol 2000	Polymer	7% (*w*/*v*)	+

**Table:** Values in parentheses are for the outer shell.

Parameter	HEWL–NaCMC	HEWL–grease	GI–F-127	GI–grease	TRL–NACMC†
Nozzle diameter (µm)	150	100	100	100	150
SLS beamline	PXII	PXII	PXII	PXII	PXII
Wavelength (Å)	1.033	0.954	1.033	1.033	1.033
Space group	*P*4_3_2_1_2	*P*4_3_2_1_2	*I*222	*I*222	*P*6_1_22
Unit-cell parameters, *a*, *b*, *c* (Å)	79.0, 79.0, 38.1	79.0, 79.0, 38.1	93.0, 98.6, 101.8	93.0, 98.7, 101.9	93.0, 93.0, 130.0
α, β, γ (°)	90, 90, 90	90, 90, 90	90, 90, 90	90, 90, 90	90, 90, 120
No. collected images	26310	23818	24412	28084	62926
Hit rate (%)	36	91	86	57	91
No. crystal hits/indexed images‡	9472/5362	21715/5348	20961/8603	16122/8593	57226/4556
Indexing rate§ (%)	57	25	41	57	8
Resolution range	25.0–1.9	25.0–1.9	25.0–2.0	25.0–2.0	25.0–2.3
Completeness (%)	99.9 (100)	99.9 (100)	99.9 (100)	99.9 (100)	99.9 (100)
Multiplicity	328.5 (262.3)	252.2 (180.7)	197.1 (119.5)	185.8 (112.8)	570.4 (470.3)
*I*/σ(*I*)	11.0 (5.1)	9.5 (8.6)	5.3 (1.7)	5.1 (2.6)	10.0 (2.2)
*R*_mrgd-F_	18.3 (47.7)	15.1 (17.3)	31.5 (123.9)	27.2 (70.2)	23.6 (104.7)
CC_1/2_	94.4 (84.8)	87.7 (89.9)	90.8 (48.2)	81.2 (50.9)	96.9 (74.9)
Overall Wilson *B* factor (Å^2^)	29.9	20.2	28.1	22.4	40.3

**Table:** 

Parameter	TRL–grease	TRL–F-127	TRL–grease	BR–LCP–F-127	BR–LCP
Nozzle diameter (µm)	150	100	100	100	100
SLS beamline	PXII	PXII	PXII	PXII	PXII
Wavelength (Å)	1.033	1.033	1.033	1.033	1.033
Space group	*P*6_1_22	*P*6_1_22	*P*6_1_22	*P*6_3_	*P*6_3_
Unit-cell parameters, *a*, *b*, *c* (Å)	93.0, 93.0, 130.0	93.0, 93.0, 130.2	93.1, 93.1, 130.0	62.2, 62.2, 110.7	62.2, 62.2, 109.6
α, β, γ (°)	90, 90, 120	90, 90, 120	90, 90, 120	90, 90, 120	90, 90, 120
No. collected images	20456	11188	18156	13285	42457
Hit rate (%)	94	55	58	93	98
No. crystal hits/indexed images‡	19222/4529	6124/4549	10533/4529	12383/4229	41701/4218
Indexing rate§ (%)	24	74	43	34	10
Resolution range	25.0–2.0	25.0–2.0	25.0–2.0	25.0–2.3	25.0–2.3
Completeness (%)	99.9 (100)	99.9 (100)	99.9 (100)	99.9 (100)	99.9 (100)
Multiplicity	241.2 (154.9)	353.3 (217.8)	277.3 (167.8)	149.3 (106.4)	191.6 (130.7)
*I*/σ(*I*)	4.9 (3.3)	5.3 (0.9)	5.3 (2.3)	6.5 (2.8)	6.5 (1.9)
*R*_mrgd-F_	23.4 (48.4)	43.3 (242.7)	31.0 (89.1)	24.3 (73.5)	28.9 (120.1)
CC_1/2_	82.3 (59.8)	92.6 (42.5)	84.8 (50.5)	94.3 (67.6)	94.9 (62.1)
Overall Wilson *B* factor (Å^2^)	16.4	34.0	22.2	42.2	47.8

† A similar number of randomly chosen images were taken for comparison.

‡ Worse statistics due to the very high stream velocity required for stable flow under the conditions of the experiment.

§ Differences in indexing rates are related to sample preparation. A too-high crystal concentration in the sample resulted in many multiple lattices containing diffraction images in the data set (as manifested by a very high hit rate). These were not always indexed by *nXDS*, thus giving a low indexing rate.
