# Supplementary material for: Viscous hydrophilic injection matrices for serial crystallography
Source: IUCrJ. 2017 May 5;4(Pt 4):400–10. doi: 10.1107/S2052252517005140 (PMC5571803; doi:10.1107/S2052252517005140)
Supplement: Supplementary file 1 [file m-04-00400-sup1.pdf]

IUCrJ

Volume 4 (2017)

Supporting information for article:

Viscous hydrophilic injection matrices for serial crystallography

Gabriela Kovácsová, Marie Luise Grünbein, Marco Kloos, Thomas R. M. Barends, Ramona Schlesinger, Joachim Heberle, Wolfgang Kabsch, Robert L. Shoeman, R. Bruce Doak and Ilme Schlichting

Supplementary Movies S1-S4.

Movie S1.

Close-up view on the extruding stream from the HVE injector nozzle with the online microscope camera at beamline X10SA (PXII) at the SLS. The size of the X-ray beam is 50 × 10 µm. The ID of the nozzle is 150 µm and the extruding stream consists of HEWL crystals embedded in NaCMC.

Movie S2

Close-up view on the extruding stream from the HVE injector nozzle with the online microscope camera at beamline X10SA (PXII) at the SLS. The size of the X-ray beam is 50 × 10 µm. The ID of the nozzle is 100 µm and the extruding stream consists of TRL crystals embedded in F-127.

Movie S3

Close-up view on the extruding stream from the HVE injector nozzle with the online microscope camera at beamline X10SA (PXII) at the SLS. The size of the X-ray beam is 50 × 10 µm. The ID of the nozzle is 100 µm and the extruding stream consists of bR crystals in LCP mixed with F-127.

Movie S4

Close-up view on the extruding stream from the HVE injector nozzle with the online microscope camera at beamline X10SA (PXII) at the SLS. The size of the X-ray beam is 50 × 10 µm. The ID of the nozzle is 100 µm and the extruding stream consists of bR crystals in LCP without F-127. While the diameter of the stream is fairly constant, the stream velocity is not. When the stream flows slowly, crystals (purple plates) extruding downstream can be clearly identified. When the stream accelerates, extruded crystals cannot be seen clearly and appear vertically “smeared”.

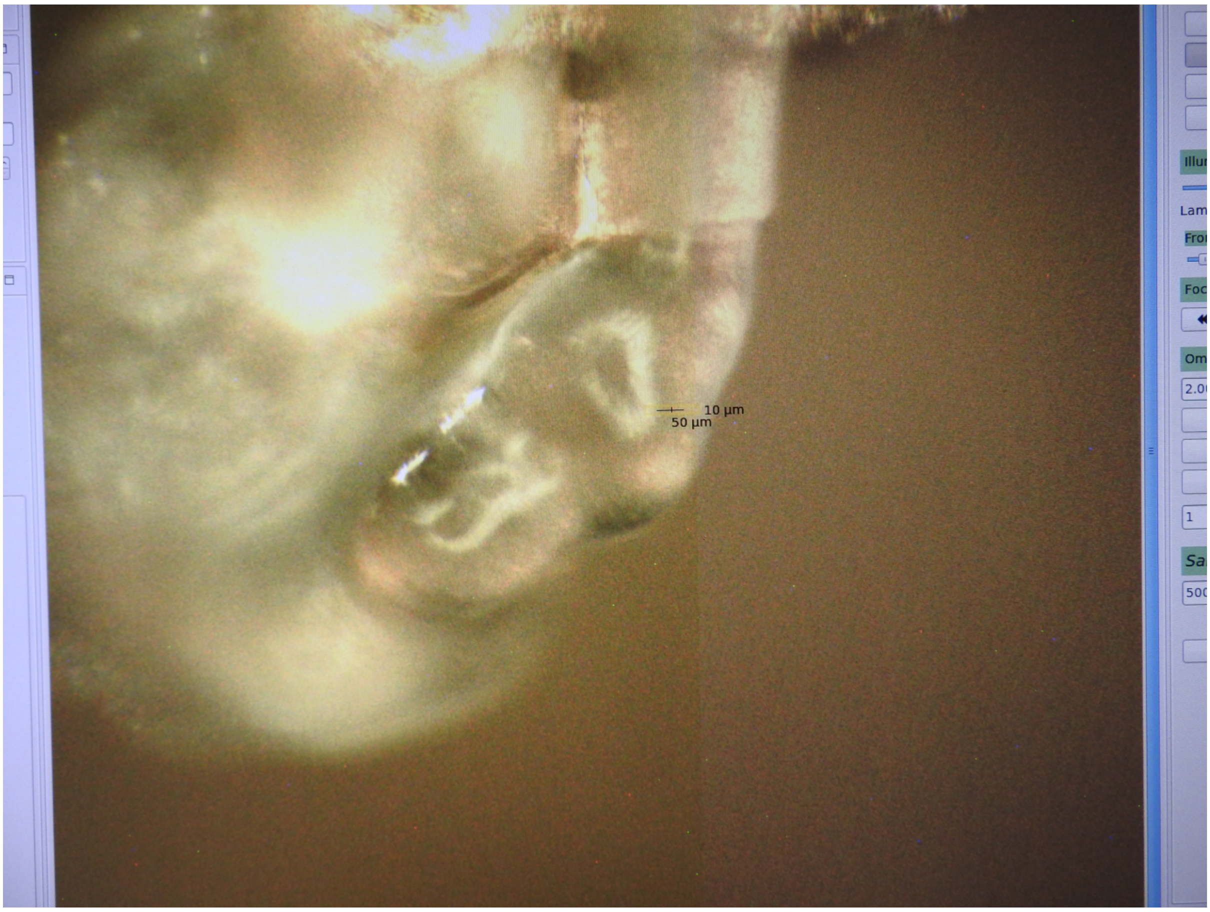

Figure S1 Close-up view of the extruded grease sample with embedded TRL crystals. The continuous flow of the grease matrix was disturbed by sudden “curling up” and attaching of the grease to the tip of the inner and outer capillary. This typically clogs the outer gas capillary and thorough cleaning of both capillaries is needed.
